# Human salivary protein-derived peptides specific-salivary SIgA antibodies enhanced by nasal double DNA adjuvant in mice play an essential role in preventing *Porphyromonas gingivalis* colonization: an in-vitro study

**DOI:** 10.1186/s12903-023-02821-6

**Published:** 2023-02-24

**Authors:** Kayo Koyanagi, Kosuke Kataoka, Hideki Yoshimatsu, Kohtaro Fujihashi, Tatsuro Miyake

**Affiliations:** 1 Department of Preventive and Community Dentistry, Graduate School of Dentistry, Osaka Dental University, 1-8 Kuzuha Hanazono-Cho, Hirakata-Shi, Osaka, 573-1121 Japan; 2 Department of Oral Health Science and Social Welfare, Graduate School of Oral Sciences, Tokushima University, 3-18-15 Kuramoto-Cho, Tokushima-Shi, Tokushima, 770-8504 Japan; 3 Department of Human Mucosal Vaccinology, Chiba University Hospital, Research Institute of Disaster Medicine, Chiba University, 1-8-1 Inohana, Chuo-Ku, Chiba-Shi, Chiba, Japan; 4 Division of Mucosal Vaccine, International Vaccine Design Center, The Institute of Medical Science, The University of Tokyo, 4-6-1, Shiroganedai, Minato-Ku, Tokyo, 108-8639 Japan; 5 Department of Pediatric Dentistry, School of Dentistry, The University of Alabama at Birmingham, 1919 7Th Avenue South, Birmingham, AL 35233 USA

**Keywords:** Statherin, Acidic proline-rich protein 1 (PRP1), *Porphyromonas gingivalis* (Pg), Colonization, Salivary secretory IgA antibody (Salivary SIgA Ab)

## Abstract

**Background:**

We previously showed that fimbriae-bore from *Poryphyromonas gingivalis* (Pg), one of the putative periodontopathogenic bacteria specifically bound to a peptide domain (stat23, prp21) shared on statherin or acidic proline-rich protein 1 (PRP1) molecule of human salivary proteins (HSPs). Here, we investigated whether the nasal administration of DNA plasmid expressing Flt3 ligand (pFL) and CpG oligodeoxynucleotide 1826 as double DNA adjuvant (dDA) with stat23 and prpr21 induces antigen (Ag)-specific salivary secretory IgA (SIgA) antibodies (Abs) in mice. Further, we examined that stat23- and prpr21-specific salivary SIgA Abs induced by dDA have an impact on Pg-binding to human whole saliva-coated hydroxyapatite beads (wsHAPs).

**Material and methods:**

C57BL/6N mice were nasally immunized with dDA plus sta23 or/and prp21 peptide as Ag four times at weekly intervals. Saliva was collected one week after the final immunization and was subjected to Ag-specific ELISA. To examine the functional applicability of Ag-specific SIgA Abs, SIgA-enriched saliva samples were subjected to Pg binding inhibition assay to wsHAPs.

**Results:**

Significantly elevated levels of salivary SIgA Ab to stat23 or prp21 were seen in mice given nasal stat23 or prp21 with dDA compared to those in mice given Ag alone. Of interest, mice nasally given the mixture of stat23 and prp21 as double Ags plus dDA, resulted in both stat23- and prp21-specific salivary SIgA Ab responses, which are mediated through significantly increased numbers of CD11c^+^ dendritic cell populations and markedly elevated Th1 and Th2 cytokines production by CD4^+^ T cells in the mucosal inductive and effector tissues. The SIgA Ab-enriched saliva showed significantly reduced numbers of live Pg cells binding to wsHAPs as compared with those in mice given double Ags without dDA or naïve mice. Additionally, saliva from IgA-deficient mice given nasal double Ags plus dDA indicated no decrease of live Pg binding to wsHAPs.

**Conclusion:**

These findings show that HSP-derived peptides-specific salivary SIgA Abs induced by nasal administration of stat23 and prp21 peptides plus dDA, play an essential role in preventing Pg attachment and colonization on the surface of teeth, suggesting a potency that the SIgA may interrupt and mask fimbriae-binding domains in HSPs on the teeth.

**Supplementary Information:**

The online version contains supplementary material available at 10.1186/s12903-023-02821-6.

## Introduction

Bacterial colonization starts with the adherence of the microbe to the surface of the mucosa and skins, subsequently leading to the establishment of infection. Also in the oral cavity, the adherence of oral bacteria to the surface of the oral mucosa and tooth is the first step in colonization, and therefore represents a critical virulence factor. Periodontitis is an infection-driven disease induced by microbial biofilm [1] and is one of the most prevalent infectious diseases worldwide, which is characterized by the destruction of periodontal supportive tissues, including inflammation of the gingiva and alveolar bone loss [2]. In the new periodontitis classification scheme, periodontitis was characterized based on four stagings by the severity of the disease as well as the complexity of disease management and three grades by the risk of periodontitis progression. In addition, the conditions of smoking and diabetes are also considered in the decision of the grade [3]. *Porphyromonas gingivalis* (Pg) is a gram-negative anaerobic bacterium that forms black-pigmented colonies and is involved in the periodontitis progression [4]. Recent works have suggested that the fimbriae of Pg is closely associated with the pathogenesis or progression of Alzheimer’s disease [5] and atherosclerosis [6] as well as several types of periodontal diseases [7]. Inhibition of Pg colonization of the surface of teeth is one of important strategies for preventing Pg infection of the oral cavity. In this regard, Pg fimbriae play a key role in the initial bacterial adherence and colonization of the surface of oral mucosa and teeth; Pg adheres to and colonizes oral mucosa and teeth by interactions between fimbriae and certain salivary proteins contained in pellicles [8]. We have previously demonstrated that Pg fimbriae exhibit specific and strong binding to acidic proline-rich protein 1 (PRP1; a 150-amino-acid-long molecule) and statherin (a 43-amino-acid-long molecule) of the human salivary proteins (HSPs) on the solid phase through protein–protein interactions [9]. Furthermore, we have identified minimal amino acid sites within the PRP1 and statherin sequences that are sufficient to permit in vitro binding to Pg fimbrillin (FimA), a subunit protein of Pg fimbriae; specifically, a YTF sequence in statherin or a PQ sequence in PRP1 appear to be key amino acid sequences for the binding of FimA to PRP1 or statherin immobilized on the hydroxyapatite beads [10, 11]. In addition, it has been proposed that peptides corresponding to the FimA-binding sites of PRP1 and statherin might serve as the basis of oral vaccination (a type of passive immunization-type mucosal vaccine) intended to prevent Pg colonization [11, 12].

To efficiently elicit immune responses to antigens (Ags) in various mucosal and systemic lymphoid tissues, active immunization-type mucosal vaccines require suitable adjuvants [13]. In this regard, we have shown that a DNA plasmid expressing the Flt3 ligand (pFL) as a mucosal adjuvant preferentially increases CD8^+^ dendritic cells (DCs) in the mucosal tissues and subsequently elicits Ag-specific Th2-type mucosal immune responses mediated by IL-4, inducing the production of CD4^+^ T cells when mice were nasally immunized with a combination of ovalbumin (OVA) or bacterial Ag plus pFL [14, 15]. Furthermore, we have shown that nasal administration of a double DNA adjuvant (dDA) consisting of CpG oligodeoxynucleotide 1826 (CpG ODN), a known inducer of Th1-type immune responses, in combination with pFL, elicits mucosal and systemic antibody (Ab) responses to OVA [16], pneumococcal surface protein A (PspA) [17], influenza virus hemagglutinin [18], and recombinant FimA (rFimA) [19], through DCs activation [20]. In addition, we previously have shown that nasal administration of rFimA, in combination with dDA, effectively reduced Pg infection in the respiratory tract; this efficacy was mediated by increased numbers of CD8^+^ and CD11b^+^ DCs and balanced Th1- and Th2-type cytokine responses in the mucosal tissues [21]. Furthermore, phosphorylcholine-specific IgM Ab, induced by dDA through interactions between DCs and B-1 B cells in the spleen, peritoneal cavity, and nasopharyngeal associated-lymphoid tissues (NALT) inhibited the progression of atherogenesis [22].

It is well-known that secretory IgA (SIgA) Ab is the major isotype, which is secreted primarily as dimeric or polymeric forms in the mucosal environment, such that human whole saliva contains SIgA, IgG, and IgM Abs at levels of approximately 200, 2 and 1 mg/L respectively [23]. SIgA Abs play a role as the body’s first line of defense by neutralizing viruses and toxins and inhibiting bacterial adherence to host mucosal surfaces [24]. Given the presence of SIgA Abs in human external secretions and the fact that SIgA Abs display more Ag-binding sites than do IgG Abs, SIgA Abs effectively bind to a variety of bacterial Ags and auto-Ags [25]. Furthermore, the intrinsic resistance of SIgA Abs to proteolysis by various bacterial enzymes, which is enhanced by association with the secretory component [26], is of functional vantage in external secretions, particularly those of the gastrointestinal tract and oral cavity, environments that are rich in proteolytic enzymes. Mucosal SIgA Abs inhibit the absorption of soluble and particulate Ags from mucosal surfaces by forming large immune complexes [27]. Furthermore, endogenous commensal gut bacteria are coated in vivo with corresponding Abs that, in turn, preclude the adherence of the microbes to epithelial cell receptors [28, 29]. In addition, SIgA Abs have also been shown to provide direct protection from relevant viral or bacterial Ags in murine models [27, 30]. In those experiments, SIgA Abs were applied directly onto a mucosal surface by instillation into the respiratory tract, including the oral cavity, or into the gastrointestinal tract, prior to or concomitant with the infectious inoculum.

In terms of systemic and mucosal immune responses to Pg infection, serum levels of anti-Pg IgG Abs in adult and early-onset periodontitis patients have been shown to be higher than those in healthy subjects; it was moreover shown that these Ab responses exhibit protective roles in the disease process [31]. Furthermore, IgA Abs specific for periodontal pathogenic microorganisms in the gingival crevicular fluid have been shown to play a protective role in the onset of periodontal disease [32]. On the other hand, it also has been reported that healthy individuals possess higher serum levels of Pg-specific IgG Abs compared to patients with periodontal disease [33]. We have also shown that salivary SIgA Abs to FimA induced by nasal dDA block Pg binding to a salivary protein on the hydroxyapatite beads (HAPs) [19]. However, despite these studies based on systemic and mucosal immune responses to Pg infection, the proposed protective roles of IgG and IgA Abs in periodontal disease remain unclear and unconfirmed. In the present study, we examined whether nasal administration of Ags with dDA could induce salivary SIgA Abs to stat23 or prp21 (HSPs-derived peptides) in mice. In addition, we assessed the ability of peptides-specific SIgA Abs to block adherence of live Pg cells to human whole saliva-coated HAPs (wsHAPs), an experimental model of the intraoral tooth surface.

## Materials and methods

### Animals

All mice in this study were 6–8 weeks of age. Specific pathogen-free female C57BL/6N (IgA^+/+^) mice were obtained from SLC Japan, Hamamatsu, Japan. IgA-deficient (IgA^−/−^) female mice (IgA^−/−^ mice; C57BL/6 genetic background) [34] were kindly provided by Dr. Tomoko Kurita-Ochiai Department of Microbiology and Immunology, Nihon University School of Dentistry at Matsudo. Upon arrival, animals were maintained at up to five mice per cage in horizontal laminar flow cabinets and were provided ad libitum with sterile food and water as part of a specific pathogen-free facility at Osaka Dental University (Hirakata, Japan). This study conformed the ARRIVE guidelines.

### Purification of human saliva proteins and preparation of synthetic peptides

Stimulated saliva was collected from a 27-year-old female donor; PRP1 were purified as described by Ramasubbu et al. [35], and then frozen and preserved at < −20 °C until use. Statherin (43 amino acid in length) and HSP-derived peptides (stat23: YQPVPEQPLYPQPYQPQYQQYTF; prp21: PQGPPPQGGRPQGPPQGQSPQ) with the indicated peptide sequences were synthesized by FASMAC Co. Ltd. (Kanagawa, Japan) and provided at > 99% purity.

### Nasal adjuvants

pFL consists of the pUNO-mcs vector plus the full-length murine *Flt3 ligand* cDNA (InvivoGen, San Diego, CA, USA). The plasmid was purified using the EndoFree Plasmid Giga kit (QIAGEN, Valencia, CA, USA) [19, 21, 22]. The lipopolysaccharide content of the purified pFL was assessed using the Limulus amebocyte lysate assay (BioWhittaker, Walkersville, MD, USA) demonstrating that LPS was present at levels of < 0.1 endotoxin units per μg of plasmid. A synthetic oligodepxynucleotide containing CpG motif (CpG ODN) 1826 was obtained from FASMAC Co., Ltd., for use as the nasal adjuvant.

### Schedule for nasal immunization and saliva sample collection

**Fig. 1:**
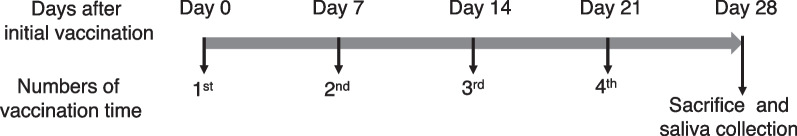
Schedule of nasal vaccination and saliva sampling. Groups of C57BL6/N mice (N = 5 mice) were nasally immunized with HSPs-derived peptide Ags with or without dDA 4 times at weekly intervals for four consecutive weeks. Saliva was collected from the mice in each at 7 days after the final immunization

Mice were nasally immunized with 6 µL/nostril phosphate-buffered saline (PBS) containing 50 μg of stat23 and/or prp21 plus 50 μg of pFL and 10 μg of CpG ODN as mucosal adjuvant (dDA) four times at weekly intervals (Fig. 1). Other groups of mice were immunized nasally with 50 μg of stat23 and/or prp21 alone or dDA alone as negative controls. All mice were immunized under intraperitoneal anesthesia consisting of hydrochloric acid medetomidine (0.3 mg/kg), midazolam (4 mg/kg) and butorphanol tartrate (5 mg/kg). Saliva samples were collected seven days after the last nasal immunization and were obtained from mice following intraperitoneal injection of 100 μg of a sterile solution of pilocarpine in PBS as described previously [17, 19] (Fig. 1).

### Ag-specific IgA Ab responses analysis

To assess stat23- or prp21-specific IgA Ab levels, saliva samples were collected from mice 7 days after the last immunization and then subjected to enzyme-linked immunosorbent assay (ELISA) as described previously [19, 36]. Briefly, 96-well microtest assay plates (BD Biosciences, Oxnard, CA, USA) were coated with 1 μg/mL of stat23 or prp21 in PBS. After incubating serial dilutions of samples, horseradish peroxidase-conjugated goat anti-mouse IgA Ab (Southern Biotechnology Associates Inc., Birmingham, AL, USA) was added to the wells. The color reaction was developed using 2,2′-azino-bis (3-ethylbenzothiazoline-6-sulphonic acid) substrate buffer for 15 min at room temperature. Endpoint titers were expressed as the reciprocal log_2_ of the last dilution that gave an OD_415_ of 0.1 greater than background. Subsequently, mice were euthanized by cervical spine fracture dislocation under inhaled isoflurane anesthesia, and mononuclear cells were isolated from NALT, periglandular lymph nodes (PGLNs), nasal passages (NPs), submandibular glands (SMGs), sublingual glands (SLGs), and parotid glands (PGs) seven days after the final immunization. The mononuclear cells were then subjected to an enzyme-linked immunospot (ELISPOT) assay to enumerate the numbers of stat23- or prp21-specific IgA Ab-forming cells (AFCs) [17, 19, 36]. In brief, mononuclear cells from PGs, SLGs, and SMGs were isolated by combination of an enzymatic dissociation procedure with collagenase type IV (0.5 mg/mL; Merck KGaA, Darmstadt, Germany) followed by discontinuous Percoll® (Amersham Biosciences, Arlington Heights, IL, USA) gradient centrifugation [17, 36, 37]. PGLNs were removed aseptically and the cells were then isolated employing a mechanical dissociation method using gentle teasing through stainless steel screens as described previously [19, 37]. For isolation of mononuclear cells from NPs and NALT, a modified dissociation method was used based upon a previously described protocol [14]. Ninety-six-well nitrocellulose plates (Millititer HA; Millipore, Bedford, MA, USA) were coated with 1 μg/mL solution of stat23 or prp21 for analysis of anti-Ag-specific IgA AFCs.

### Flow cytometric analysis for CD11c^+^ DCs populations

Previous studies have reported that dDA consisting of pFL and CpG ODN promotes the induction of DCs in mucosal and systemic lymphoid tissues [16–22]. Therefore, mononuclear cells were isolated from NALT, PGLNs, NPs, SMGs, SLGs, and PGs were harvested one week after the last immunization of mice administered both stat23 and prp21 (double Ags) with or without dDA. Subsequently, the cells were stained with Brilliant Violet (BV) 421-conjugated anti-mouse CD11c monoclonal antibodies (mAbs) (BioLegend, San Diego, CA, USA). These samples then were subjected to flow cytometric analysis using a FACSVerse instrument equipped with Flow Jo software (BD Biosciences, San Jose, CA, USA).

### Cytokine production by stat23- and prp21-stimulated CD4^+^ T cells

CD4^+^ T cells were purified from NALT, PGLNs, and NPs harvested from mice immunized double Ags with or without dDA; purification was performed using an automatic cell sorter (AutoMACS®) system (Miltenyi Biotec B.V. & Co. KG, Bergisch Gladbach, Germany) as described previously [13, 17]. The purified CD4^+^ T cell fraction (> 97% CD4^+^ and > 99% viable) was resuspended at 4 × 10^6^ cells/mL in “complete medium” consisting of RPMI 1640 (Sigma-Aldrich) supplemented with 4-(2-hydroxy ethyl)-1-piperazine ethane sulphonic acid (HEPES) buffer (10 mM), L-glutamine (2 mM), nonessential amino acid solution (10 μL/mL), sodium pyruvate (10 mM), penicillin (100 U/mL), streptomycin (100 μg/mL), gentamycin (80 μg/mL), and 10% fetal calf serum. The resulting suspensions were cultured in the presence of T cell-depleted, complement-, and mitomycin-treated splenic Ag-presenting cells isolated from naïve C57BL/6N mice; these cultures were cultivated in the presence or absence of stat23 and prp21 (each 1 μg/mL). The culture supernatants were collected on Day 5 and analyzed using IFN-γ-, IL-2-, IL-4-, and IL-5-specific ELISA kits (Invitrogen, Carlsbad, CA, USA). The detection limits for individual cytokines were 5.3 pg/mL for IFN-γ, 2.0 pg/mL for IL-2, 4.0 pg/mL for IL-4, and 3.3 pg/mL for IL-5, respectively.

### Inhibition assay for binding of live Pg cells to statherin-coated HAPs (sHAPs) or PRP1-coated HAPs (pHAPs), and to wsHAPs

The functionality of HSP-derived peptide-specific SIgA Abs was assessed using SIgA-enriched saliva samples, prepared as follows. Saliva from naïve mice (protein concentration, 1.8 mg/mL), or from mice immunized nasally with double Ags plus dDA or double Ags alone (protein concentration, 1.7 mg/mL in both groups) was applied to a protein G affinity mini-column (Protein G HP SpinTrap; Cytiva, Tokyo, Japan, Immobilized MBP Column; ThermoFisher Scientific K.K., Tokyo, Japan) according to the manufacture’s protocol; the flowthrough fraction then was applied to the equivalent column from an IgM purification kit (ThermoFisher Scientific, Tokyo, Japan) according to the manufacturer’s protocol. The resulting flowthrough fraction was dialyzed overnight against KCl buffer (consisiting of 50 mM KCl, 1 mM KH_2_PO_4_, 1 mM CaCl_2_, and 0.1 mM MgCl_2_, pH 6.7) using a 10-kD cutoff Slide-A-Lyzer dialysis cassette (ThermoFisher Scientific K.K.). The dialyzed saliva was used as the SIgA-enriched saliva sample in an assay measuring the inhibition of binding of Pg cells to sHAPs, pHAPs, or wsHAPs. Briefly, 3 mg of HAPs were placed in a siliconized tube and incubated with 100 µL of statherin (100 µg/mL), PRP1 (100 µg/mL), or human whole saliva (protein concentration, 1.6 mg/mL) overnight at room temperature. HAPs of each type then were washed three times with KCl buffer, and 100 µL of SIgA-enriched mouse saliva (as an inhibitor) or KCl buffer (as a control) were added to the HAPs; these mixtures were incubated at room temperature for 1 h. After washing of the HAPs three times with KCl buffer, 100 μL (1 × 10^8^ cells) of live Pg cells were added to each tube, and the mixtures were incubated at room temperature for 1 h. Subsequently, to remove excess unbound Pg, the HAPs were soaked to Percoll® (Cytiva, Tokyo, Japan) and were transferred to individual wells of an opaque 96-well plate. The number of live Pg cells bound to the HAPs in each well was assessed based on quantitation of adenosine triphosphate (ATP) generated by live Pg cells, as detected by measuring luciferase mediated luminescence using a Bac Titer-Glo Microbial cell viability assay (Promega Co., Madison, WI, USA). All assays were performed in triplicate on three separate occasions.

### Statistical analysis

The data are expressed as the median and interquartile range (the first quartile—1.5 IQR, the 3rd quartile + 1.5 IQR), or as the mean ± standard error (SE). All mouse groups were compared to control mice via two-tailed unpaired Mann–Whitney U-tests or Student’s t-tests using GraphPad Prism softwere (version 7 GraphPad, San Diego, CA, USA). *p* values of < 0.05 was considered statistically significant, and *p* values are indicated as *p < 0.05 and **p < 0.01.

## Results

### Enhancement of salivary stat23/prp21-specific SIgA Ab responses

**Fig. 2:**
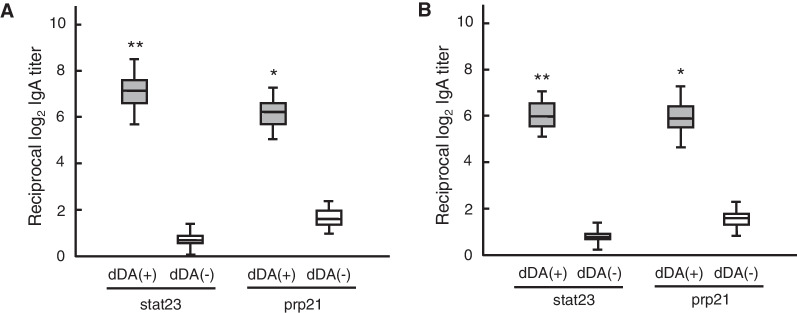
Salivary SIgA Ab responses to stat23 or prp21 peptide in mice. (**A**) Salivary SIgA Ab responses to stat23 or prp21 in mice given nasally single Ags with/without dDA. (**B**) Salivary stat23 or prp21 specific-SIgA Ab in mice given nasally double Ags with/without dDA. The levels of anti-peptide SIgA Abs in saliva were determined by the respective peptide Ag-specific ELISA assay. The values are presented as the median and interquartile range (the first quartile—1.5 IQR, the 3rd quartile + 1.5 IQR) of 15 mice in each group and were compared via two-tailed non-paired Mann–Whitney U-tests. *p < 0.05, **p < 0.01 vs. mice immunized with peptide Ag alone without dDA (dDA (-))

Antigen-specific SIgA Ab production was examined by ELISA using saliva from mice administered nasally stat23 or prp21 as an Ag, in combination with dDA consisting of pFL and CpG ODN. As shown in Fig. 2A, nasal immunization with stat23 or prp21 plus dDA resulted in significantly increased levels of Ag-specific SIgA Ab in the saliva compared to the levels in the saliva of mice administered nasally with Ag alone. No stat23- or prp21- specific SIgA Ab responses were seen in the saliva of non-immunized (naïve) mice or that of mice administered dDA alone (data not shown). Next, mice were immunized nasally with both stat23 and prp21 (double Ags) with/without dDA. As with nasal administration of the single Ags (stat23 or prp21, with dDA), significantly higher levels of salivary Ag-specific SIgA Ab were seen in mice administered with double Ags plus dDA compared to those in mice administered with double Ags alone (Fig. 2B).

### Augmented SIgA AFCs in mucosal inductive and effective lymphoid tissues of mice administered stat23 and/or prp21 plus dDA

The numbers of stat23- or prp21-specific IgA Ab producing cells were assessed by ELISPOT assay. As expected, elevated numbers of stat23- or prp21-specific IgA AFCs were detected in NALT, PGLNs, NPs, PGs, SMGs and SLGs of mice administered nasal stat23 or prp21 Ag plus dDA (Table 1) compared to those of mice administered nasally stat23 or prp21 Ag alone. In addition, the numbers of Ag-specific IgA AFCs in NALT, NPs, SMGs and SLGs of mice administered nasally with stat23 or prp21 plus dDA were significantly elevated compared to those of mice immunized nasally with stat23 or prp21 alone. These results clearly showed that use of the novel dDA for nasal vaccination effectively elicits the respective peptide-specific SIgA Ab responses in the salivary gland-associated lymphoid tissues and nasal mucosa in mice (Table 1). Interestingly, among the major salivary glands of mice immunized nasally with stat23 or prp21 plus dDA, the higher numbers of stat23- or prp21-specific IgA AFCs were detected in SLGs than in PGs and SMGs (Table 1). Since nasal immunization with dDA may induce systemic immunity (as demonstrated previously by mucosal immune responses to the protein Ags [16, 17]), stat23- or prp21-specific Ab responses in plasma and spleen were examined. Nasal administration of stat23 or prp21 plus dDA elicited significantly elevated levels of stat23- or prp21-specific IgG and IgA Ab responses in the plasma and spleen of immunized mice compared to those in mice administered nasal stat23 or prp21 alone (data not shown). Taken together, our results showed that the use of dDA effectively enhances mucosal IgA and systemic IgG and IgA Ab responses to peptides derived from HSPs. Next, we tested whether simultaneous nasal application of the mixture of stat23 and prp21 plus dDA provoked stat23- and prp21- specific SIgA Ab responses in oral- and nasal-associated lymphoid tissues. Significantly elevated numbers of stat23- and prp21-specific IgA AFCs were seen in NALT, PGLNs, NPs, SMGs, SLGs, and PGs of mice given nasally simultaneous the double Ags with dDA, compared to those in mice administered nasally double Ags without dDA. (Table 2, Additional file 1: Fig. S1). Also, of three major salivary glands (SMGs, SLGs, and PGs), the SLGs displayed the highest numbers of stat23- or prp21-specific IgA AFCs (Table 2, Additional file 1: Fig. S1). These findings indicated that dDA potentiates mucosal responses to the respective Ags, even when the different Ags are applied simultaneously by the intranasal route.

**Table 1:** The numbers of HSPs-derived stat23- or prp21-specific SIgA AFCs in mice administered nasally single Ag (stat23 or prp21) with/without dDA

Mucosal inductive or effective tissues		Given prp21Ags			stat23	
		stat23-Specific SIgA AFCs				prp21-specific SIgA AFCs (in 10^6^ mononuclear cells) (in 10^6^ mononuclear cells)
		dDA	+	−	+	−
Salivary glands	NALT		*14.6 ± 0.9	4.5 ± 0.5	*17.8 ± 1.5	7.8 ± 0.2
	PGLNs		8.4 ± 1.3	4.1 ± 0.5	9.2 ± 0.7	5.9 ± 0.8
	NPs		*162 ± 23	16 ± 4.6	*132 ± 27	23 ± 5.4
	SMGs		*16.9 ± 3.3	5.4 ± 1.3	*25.1 ± 4.9	6.6 ± 1.7
	SLGs		*25.8 ± 4.3	14.7 ± 5.9	*35.3 ± 5.7	12.7 ± 4.8
	PGs		9.8 ± 3.1	5.4 ± 0.8	8.2 ± 3.0	5.1 ± 0.6

**Table 2:** The numbers of HSPs-derived stat23- or prp21-specific SIgA AFCs in mice given nasally double Ags (stat23 and prp21) with/without dDA

Mucosal inductive or effective tissues		Given stat23 + prp21			stat23 + prp21Ags	
		stat23-Specific SIgA AFCs				prp21-specific SIgA AFCs(in 10^6^ mononuclear cells) (in 10^6^ mononuclear cells
		dDA	+	−	+	−
	NALT		*9.9 ± 2.3	3.1 ± 0.9	*12.2 ± 2.5	3.8 ± 1.8
Salivary glands	PGLNs		*10.1 ± 2.8	2.1 ± 0.6	*13.3 ± 2.9	2.6 ± 1.1
	NPs		*121 ± 18	9.2 ± 2.6	*151 ± 34	12.7 ± 4.4
	SMGs		*16.7 ± 2.4	3.7 ± 0.9	*15.3 ± 2.8	2.3 ± 0.5
	SLGs		*28.7 ± 4.2	14.0 ± 1.4	*30.3 ± 3.5	15.0 ± 2.9
	PGs		*12.3 ± 3.1	2.3 ± 0.5	*14.7 ± 1.2	4.0 ± 1.4

Mice were given nasally the single peptide Ag (stat23 or prp21) with/without dDA four times at weekly intervals. Seven days after the last immunization, the mononuclear cells from the various lymphoid tissues were collected and were subjected to an ELISPOT assay. The values are presented as the means ± SE of 15 mice per group. Comparisons were performed using a two-tailed unpaired Student’s t-test vs. mice immunized with stat23 or prpr21 alone (dDA(-)), *p < 0.05.

Mice were nasally administered with the double Ags (stat23 and prp21) with/without dDA, four times at weekly intervals. Seven days after the final immunization, the mononuclear cells from the various lymphoid tissues were collected and subjected to an ELISPOT assay. The values are presented as the means ± SE of 15 mice per group. Comparisons were performed using a two-tailed unpaired Student’s t-test vs. mice immunized with double Ags alone (dDA(-)), *p < 0.05.

### Induction of CD11c^+^ DCs in mucosal lymphoid tissues

In our previous studies, nasal administration with pFL and CpG ODN as a mucosal adjuvant resulted in increased numbers of CD11c^+^ DCs which contribute to the Ag-presentation and subsequently to the induction of specific immune responses to OVA [16], recombinant PspA [17], hemagglutinin of influenza virus [18] or FimA of Pg [20]. Therefore, the frequencies of CD11c^+^ DCs in the various mucosal tissues of mice given double Ags with dDA or double Ags alone without dDA by flow cytometry analysis were examined. Nasal vaccination with double Ags plus dDA resulted in significantly increased proportions of CD11c^+^ cells in NALT, PGLNs and NPs, compared to those obtained following nasal vaccination with double Ags alone (Additional file 2: Table S1, Additional file 3: Fig. S2); notably, similar effects were not seen in the SMGs, SLGs, or PGs.

### Production of Th1- and Th2-type cytokines by mucosal CD4^+^ T cells stimulated with double Ags

Cytokine production by double Ags-stimulated CD4^+^ T cells isolated from NALT, PGLNs, and NPs of mice administered nasally with double Ags with or without dDA was tested. When CD4^+^ T cells from NALT and NPs of mice given dDA were stimulated with double Ags, significantly higher levels of IFN-γ, IL-2, IL-4, and IL-5 production were exhibited compared to those of control mice (Additional file 2: Table S2). Double Ags-stimulated CD4^+^ T cells from PGLNs of mice given double Ags plus dDA showed significant elevated levels of IFN-γ, IL-2, and IL-4 production (Additional file 4: Table S2). These results showed that nasal immunization of mice with dDA (consisting of pFL and CpG ODN) provokes a balanced Th1- and Th2-type cytokine response in mucosal inductive and effective tissues.

### Inhibition of live Pg binding to sHAPs or pHAPs by SIgA Ab-enriched saliva of mice given nasally double Ags with/without dDA

**Fig. 3:**
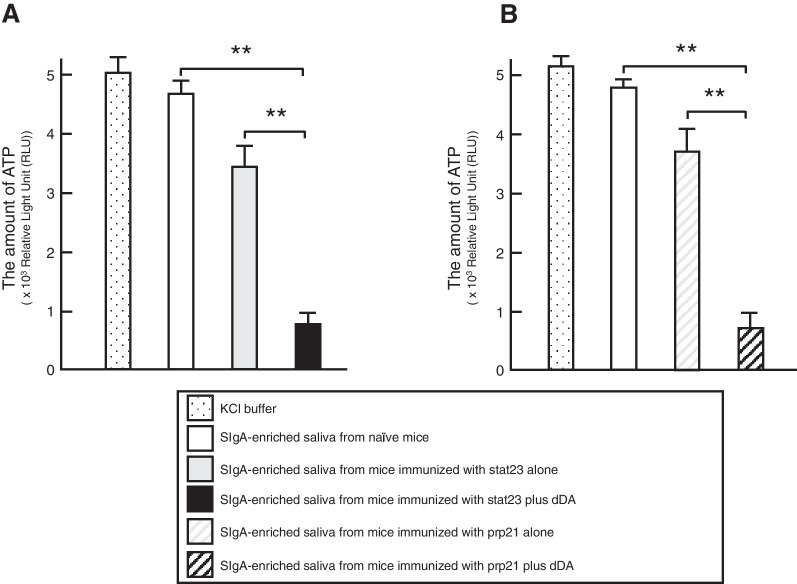
The inhibition assay for binding of live Pg cells to sHAPs incubated with SIgA Ab-enriched saliva from mice administered nasally stat23 with/without dDA (**A**) or to pHAPs with SIgA Ab-enriched saliva from mice administered nasally prp21 with/without dDA (B). SIgA Ab-enriched saliva (100 µL) (i.e., saliva from which IgM and IgG Abs were removed using affinity columns chromatography) was added to sHAPs (A) or pHAPs (**B**) in siliconized glass tubes; the resulting mixtures were incubated for 3 h at room temperature. The HAPs were washed three times with 500 µL of KCl buffer. Subsequently, Pg ATCC 33,277 (1 × 10^8^ cells in 200 µL of KCl buffer) was added to each tube. After incubation for another 3 h at room temperature, the HAPs were washed three times with 500 µL of KCl buffer and then transferred to white 96-well plates. The levels of ATP (indicative of live Pg cells bound to the HAPs) were determined using a luciferase assay and luminometer. Values are represented as the means ± SE from three replicates. Comparisons were performed by two-tailed unpaired Student’s t-test. **p < 0.01

To verify the functionality of HSP-derived peptides-specific salivary SIgA Abs in the saliva of mice given nasally stat23 or prp21 with/without dDA, inhibition assay of a live Pg cells binding was performed using sHAPs or pHAPs. The protein concentration ranges of dialyzed SIgA Ab-enriched saliva ranged from 0.9  to  1.2 mg/mL in naïve (non-immunized) mice and in mice immunized with stat23 or prp21 with/without dDA. Measurements of luminescence (detecting levels of ATP, a marker for the presence of live Pg cells attached to sHAPs showed that beads incubated with SIgA Ab-enriched saliva samples from mice immunized with stat23 plus dDA bound significantly lower numbers of live Pg cells compared to beads incubated with saliva from mice given nasal stat23 alone or those from naïve mice (Fig. 3A). Likewise, pHAPs exposed to SIgA Ab-enriched saliva samples from mice immunized with prp21 plus dDA bound significantly lower numbers of live Pg cells compared to beads incubated with saliva from mice given nasal prp21 alone or those from naïve mice (Fig. 3B).

### Inhibitory effects of Pg binding to wsHAPs by SIgA Ab-enriched saliva from mice given nasally double Ags with/without dDA and by saliva from IgA-deficient mice

**Fig. 4:**
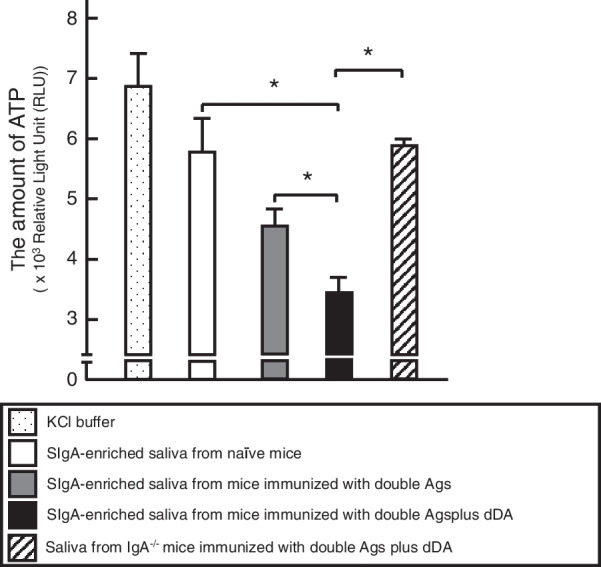
Inhibition of the binding of live Pg cells to wsHAPs by SIgA Ab-enriched saliva in wild-type (IgA^+/+^) or by saliva from IgA^−/−^ mice given nasally both stat23 and prp21 with/without dDA. An aliquot (100 µL) of SIgA Ab-enriched saliva (i.e., saliva from which IgM and IgG Abs had been removed using affinity columns chromatography) or IgA^−/−^ mice saliva samples were added to wsHAPs in the was added to wsHAPs in siliconized glass tubes; the mixtures then was incubated for 3 h at room temperature. Subsequently, the wsHAPs were washed three times with 500 µL of KCl buffer and Pg ATCC 33,277 (1 × 10^8^ cells in 200 µL of KCl buffer) was added to each tube. After incubation for another 3 h at room temperature, the HAPs were washed three times with 500 µl of KCl buffer and then transferred to white 96-well plates. The levels of ATP (indicative of live Pg cells bound to the wsHAPs) were determined using a luciferase assay and luminometer. Values are presented as the mean ± SE from three replicates. Comparisons were performed by two-tailed unpaired Student’s t-test. *p < 0.05

The binding inhibition of live Pg cells to wsHAPs by salivary SIgA Abs induced by nasal immunization with double Ags with dDA was investigated. wsHAPs incubated with SIgA Ab-enriched saliva samples from mice administered nasally with double Ags plus dDA bound significantly lower numbers of live Pg cells compared to beads incubated with saliva from mice administered nasally with double Ags alone or saliva from naïve mice (Fig. 4).

To further define the essential roles of stat23- and prp21-specific SIgA Abs induced by nasal vaccination with double Ags plus dDA, the assay testing binding of Pg cells to wsHAPs was repeated using saliva samples (dialyzed against KCl buffer) obtained from IgA-deficient (IgA^−/−^) mice that had been immunized nasally with double Ags plus dDA. Notably, beads incubated with saliva samples from IgA^−/−^ mice administered nasally with double Ags plus dDA showed Pg cells binding (as assessed by luminescence, indicative of ATP levels) statistically indistinguishable from those incubated with saliva from naïve mice, and significantly lower numbers of live Pg cells compared to those seen for beads incubated with SIgA Ab-enriched saliva samples from mice immunized nasally with double Ags plus dDA (Fig. 4).

## Discussion

We previously showed that Pg fimbriae specifically and strongly bind to statherin and PRP1 on the solid phase through protein–protein interactions; we further demonstrated that this interaction reflects binding of fimbrillin (FimA), a subunit protein of Pg fimbriae, to sites located within the C-terminal portions of statherin and PRP1 molecules [9, 11]. In addition, we defined the minimal amino acid sequences within C-terminal portions of statherin and PRP1 molecules which combines with FimA [9, 11]. We also showed that stat23 and prp21 (peptides corresponding to the C-terminal 23 amino acid residues of statherin and the C-terminal 21 amino acid residues of PRP1, respectively) inhibited the binding of fimbriae and Pg whole cells to sHAPs and pHAPs [9–12]. Based on these results, we proposed that stat23 and prp21, peptides derived from statherin and PRP1 might serve as components of candidate for the mucosal vaccines intended to attenuate Pg cell attachment to, and colonization of, the surface of teeth in the oral cavity. In other work, we have demonstrated that dDA (the combination of pFL and CpG ODN) has potential for use as an adjuvant for active immunity-type mucosal vaccines targeting mucosal DCs [16–22]. In the present study, we hypothesized that the use of dDA may enhance salivary SIgA Ab immune responses to stat23 or prp21 in mice. Our results showed significant enhancement of stat23- and prp21-specific SIgA Ab responses in the saliva of mice administered nasally both stat23 and prp21 as double Ags in the presence of dDA (Fig. 2B), as well as in the saliva of mice administered nasally stat 23 or prp21 with dDA (Fig. 2A). As expected, increased numbers of stat23 or prp21-specific AFCs, as evidenced by increases in the numbers of CD11c^+^ DCs, also were seen in the salivary glands and NALT, NPs, and PGLNs of mice immunized with nasally Ags plus dDA (S1 Table) compared to those of mice administered nasally with Ags alone (Tables 1 and 2). Of interest, higher numbers of stat23- or prp21-specific AFCs were seen in SLGs than in SMGs (among the major salivary glands) in mice given nasal dDA (Tables 1 and 2). Based on these findings, we propose that SLGs are a mucosal effector tissue in addition to SMGs, which already are known as a major mucosal effector tissue for IgA Ab production in the oral cavity [20, 37]. Furthermore, we speculate that nasal immunization with Ags plus dDA activates CD11c^+^ DCs; these DCs presumably then serve as Ag-presenting cells in the mucosal inductive tissues (i.e., NALT), leading to the activation of CD4^+^ T cells. The resulting effector CD4^+^ T cells could migrate into the mucosal effector tissues (e.g., SLGs and SMGs), producing Th1-/Th2-type cytokines and activating IgA-positive B cells to differentiate into plasma cells (Additional file 4: Table S2). We also speculate that the production of Th1- and Th2-type cytokines by CD4^+^ T cells, in both mucosal inductive and effector tissues, may contribute to the induction of Ag-specific IgA Ab responses, consistent with our previous studies showing that the use of the dDA results in balanced Th1-/Th2-type cytokine responses [16, 17].

Plasma cells adjacent to polymeric immunoglobulin receptor (pIgR)-expressing epithelial cells are known to produce polymeric IgA Abs which are transported and secreted on the surfaces of various mucosa via transcytosis [24]. This mechanism is thought to be responsible for the high concentrations of SIgA Ab present in saliva, nasal washes, and colostrum as well as in secretions of the pulmonary alveolus and intestine. Saliva contains remarkably higher levels of SIgA Abs than IgG and IgM Abs [23], and the SIgA Abs serve as front-line defensive tools in the oral cavity, contributing to the inhibition of bacterial and viral adherence to the surfaces of teeth and oral mucosa, to the neutralization of biologically active Ags, etc. This present study also showed that salivary stat23- and prp21-specific SIgA Abs, the production of which was enhanced using dDA as an adjuvant, play a key role in preventing the binding of Pg cells to sHAPs, pHAPs (Fig. 3), and wsHAPs (Fig. 4). We further showed that such stat23- and prp21-specific SIgA Abs are essential for preventing Pg binding to wsHAPs (Fig. 4). These findings suggest that HSPs-derived peptides-specific SIgA Abs (induced here by nasal immunization with stat23 and/or prp21 plus dDA) may provide attenuation of Pg attachment and colonization by masking fimbriae-binding domains on HSPs presented in solid phase on the pellicle of the tooth surface. This interpretation suggests, in turn, that SIgA Abs (passive immune therapy) have potential for use as Ab drugs intended to prevent Pg attachment and colonization on the surface of teeth in the oral cavity.

Human saliva has been reported to contain high levels of statherin (mean concentration 6.86 mg%, with a range of 1.6–14.7 mg%, and a standard deviation of 2.93 mg%) [38] and PRP1 (mean concentration 48.9 mg%, with a range of 19–80 mg% and standard deviation of14.5 mg%) [39]; reported elsewhere as 45 mg% [40], and 43 mg% [41]) in human saliva. Since the titers of anti-stat23 and -prp21salivary SIgA Abs (as well as those against statherin and PRP1) in human saliva remain (to our knowledge) unknown, we performed a pilot study to determine these values. Notably, we were unable to detect stat23- and prp21-specific SIgA Abs in human saliva (data not shown). Based on these findings, we infer that the oral cavity may exhibit immunologic tolerance to stat23 and prp21 Ags, despite the known abundance of statherin and PRP1 in saliva. Given that mouse-derived Abs exhibit inherent immunogenicity when mouse-derived Abs are applied to human body, the modification of mouse-derived Abs by genetic means has been conducted in recent years. For instance, it has been reported that a humanized Ab prevents human herpesvirus (HHV)-6B infection [42], that a completely humanized Ab purified from humanized mice neutralizes severe acute respiratory syndrome-related coronaviruses and variants [43], and that a monoclonal Ab exhibits anti-tumor effects by binding to human epidermal growth factor receptor 2 displayed on tumor cells [44]. Therefore, we are currently trying to develop a passive immune therapy that incorporates a partially or fully humanized Ab based on salivary stat23- and prp21-specific SIgA Abs derived from mice.

Periodontitis is one of the most prevalent infectious diseases on earth [2, 45, 46] and is characterized by the disruption of periodontal supportive tissues, including gingival inflammation and alveolar bone loss following periodontal-pathogenic bacterial infection and disturbance of host immunity; periodontitis also is thought to be a risk factor for multiple systemic diseases onset [47, 48], despite being detected in healthy people [49]. Given that Pg, a Gram-negative anaerobic microbe, has been implicated in the onset and progression of periodontal disease [4, 50], a strategy for blocking Pg cells attachment to, and colonization of, the surface of teeth is required for primary prevention. Based on the results of the present study and our previous work [7, 8], we conjecture that stat23 and prp21 might serve as components of the mucosal vaccine and the passive immune therapy which intended to prevent Pg attachment to the teeth and subsequent colonization.

## Conclusions

In summary, we showed that nasal inoculation with Ags combined with dDA successfully elicits elevated levels of salivary stat23- and prp21-specific SIgA Abs responses by induction of mucosal CD11c^+^ DCs and a balanced Th1/Th2 type cytokine response. Furthermore, IgA-enriched saliva from mice given nasal both stat23 and prp21 (the double Ags) in combination with dDA showed significant inhibition of Pg binding to wsHAPs compared to that obtained from mice administered nasal Ags alone. In addition, we confirmed an important function of salivary SIgA Abs in Pg attachment to wsHAPs by using saliva from IgA deficient (IgA^−/−^) mice administered nasal stat23 and prp21 plus dDA. Together, these results suggest that the interaction between fimbriae-binding domains on salivary proteins carried by the pellicle on the tooth surface and the salivary Ag-specific SIgA Abs induced by nasal HSPs-derived peptides plus dDA, may provide masking of fimbriae binding sites. Thus, the salivary Ag-specific SIgA Abs are candidates for an effective Ab drug to prevent Pg colonization; these Ags therefore might serve as the basis for passive immunity-type mucosal vaccines that could be administered into the oral cavity. Further studies will be essential to demonstrate the utility of mouse salivary SIgA Abs, especially as induced using dDA, in the development of drugs incorporating partially or fully humanized Abs.

## Supplementary Information

**Additional file 1: Fig. S1. The representative Ag-specific SIgA AFCs on the membranes of ELISPOT plates from NALT, PGLNs, NPs, SMGs, SLGs and PGs in mice given nasally double Ags with/without dDA.** Mononuclear cells from NALT, PGLNs, NPs, SMGs, SLGs and PGs were subjected to ELISPOT assay to detect numbers of Ag-specific AFCs. Ninety-six-well nitrocellulose plates (Millipore) were coated with each Ag, incubated for 20 h at 4° C and then washed extensively and blocked with 2% BSA in PBS solution. The blocking solution was discarded, and mononuclear cells (10^6^ / well) were added to wells and incubated for 4 h at 37° C in 5% CO_2_ in moist air. Goat horseradish peroxidase-conjugated anti-mouse IgA Ab was used as detection Ab. Following overnight incubation, plates were developed by adding 3-amino-9-ethylcarbazole dissolved in 0.1M sodium acetate buffer containing H_2_O_2_ to each well, and AFCs were counted with the aid of a stereomicroscope.

**Additional file 2: Table S1. Proportions of CD11c**^**+**^ **DCs in mucosal lymphoid tissues of mice given nasal double Ags with/without dDA, as assessed by flow cytometry assay.** Mice were nasally administered weekly for four consecutive weeks with the mixture of stat23 and prp21 with/without dDA. One week after the last immunization, the mononuclear cells from NALT, SMLNs, NPs, and three major salivary glands were collected and were stained with Brilliant violet 421-labeled anti-mouse CD11c monoclonal Ab. The resulting cells were subjected to flow cytometry analysis by FACSVerse^®^. The values are presented as the means ± SE of 10 mice in each group. Comparison were performed using a two-tailed unpaired Student’s *t*-test vs. mice immunized with the double Ags alone [dDA(-)], ^*^p<0.05.

**Additional file 3 : Fig. S2. The typical FACS plot and gating strategy in NALT, PGLNSs and NPs.** In FACS analysis, mononuclear cells from NALT, PGLNs and NPs were gated to lymphocytes by using the forward-and side-scatter properties and were subsequently analyzed for the populations of CD11c^+^ cells. Mononuclear cells from NALT, PGLNs, and NPs were stained with Brilliant violet 421-conjugated anti-mouse CD11c monoclonal antibody and were subjected to flow cytometric analysis by FACSVerse^®^. The graph represents typical profiles for each experimental group, and the percentages of CD11c^+^ DCs in the mononuclear cells are indicated in each graph. The dotted line indicates no stain samples.

**Additional file 4: Table S2. stat23 and prp21-induced CD4+ Th1- and Th2-type cytokines from mice given nasal double Ags with/without dDA.** Mice were nasally immunized weekly for 4 consecutive weeks with the mixture of stat23 and prp21 with/without dDA. One week after the final administration, CD4 T cells (4 x 10^6^ cells/mL) from NALT, PGLNs and NPs were cultured with stat23 and prp21 (each 1 μg/mL) in the presence of T cell-depleted splenic feeder cells (8 x 10^6^ cells/mL). The culture supernatants were harvested after 5 days incubation and analyzed by the respective cytokine-specific ELISA. The levels of each cytokine are expressed by subtracting the protein value of non-stimulated cultures from that of stimulated cultures. The values are presented as the means ± SE of three independent experiments. Each group consists of five mice. Comparisons were performed using a two-tailed unpaired Student’s *t*-test vs. mice immunized with double Ags [dDA(-)], *p<0.05.

## Data Availability

All data generated or analyzed during this study are included in this article.

## Abbreviations

Pg: *Porphyromonas gingivalis*
PRP1: Acidic proline-rich protein 1
HSP: Human salivary protein
pFL: DNA plasmid expressing Flt3 ligand
dDA: Double DNA adjuvant
SIgA: Secretory IgA
Ag: Antigen
Ab: Antibody
OVA: Ovalbumin
rFimA: Recombinant FimA
PBS: Phosphate-buffered saline
HAPs: Hydroxyapatite beads
wsHAPs: Human whole saliva-coated hydroxyapatite beads
pHAPs: PRP1-coated hydroxyapatite beads
sHAPs: Statherin-coated hydroxyapatite beads
CpG ODN: CpG oligodeoxynucleotide 1826
DCs: Dendritic cells
NALT: Nasopharyngeal associated-lymphoid tissues
SIgA: Secretory-IgA
ELISA: Enzyme-linked immunosorbent assay
PGLNs: Periglandular lymph nodes
NPs: Nasal passages
SMGs: Submandibular glands
SLGs: Sublingual glands
PGs: Parotid glands
ELISPOT: Enzyme-linked immunospot assay
AFCs: Ab-forming cells
