# Supplementary material for: Human salivary protein-derived peptides specific-salivary SIgA antibodies enhanced by nasal double DNA adjuvant in mice play an essential role in preventing Porphyromonas gingivalis colonization: an in-vitro study
Source: BMC Oral Health. 2023 Feb 24;23:123. doi: 10.1186/s12903-023-02821-6 (PMC9950703; doi:10.1186/s12903-023-02821-6)
Supplement: Supplementary file 1 — Additional file 1: Fig. S1. The representative Ag-specific SIgA AFCs on the membranes of ELISPOT plates from NALT, PGLNs, NPs, SMGs, SLGs and PGs in mice given nasally double Ags with/without dDA. Mononuclear cells from NALT, PGLNs, NPs, SMGs, SLGs and PGs were subjected to ELISPOT assay to detect numbers of Ag-specific AFCs. Ninety-six-well nitrocellulose plates (Millipore) were coated with each Ag, incubated for 20 h at 4° C and then washed extensively and blocked with 2% BSA in PBS solution. The blocking solution was discarded, and mononuclear cells (106 / well) were added to wells and incubated for 4 h at 37° C in 5% CO2 in moist air. Goat horseradish peroxidase-conjugated anti-mouse IgA Ab was used as detection Ab. Following overnight incubation, plates were developed by adding 3-amino-9-ethylcarbazole dissolved in 0.1M sodium acetate buffer containing H2O2 to each well, and AFCs were counted with the aid of a stereomicroscope. [file 12903_2023_2821_MOESM1_ESM.pdf]

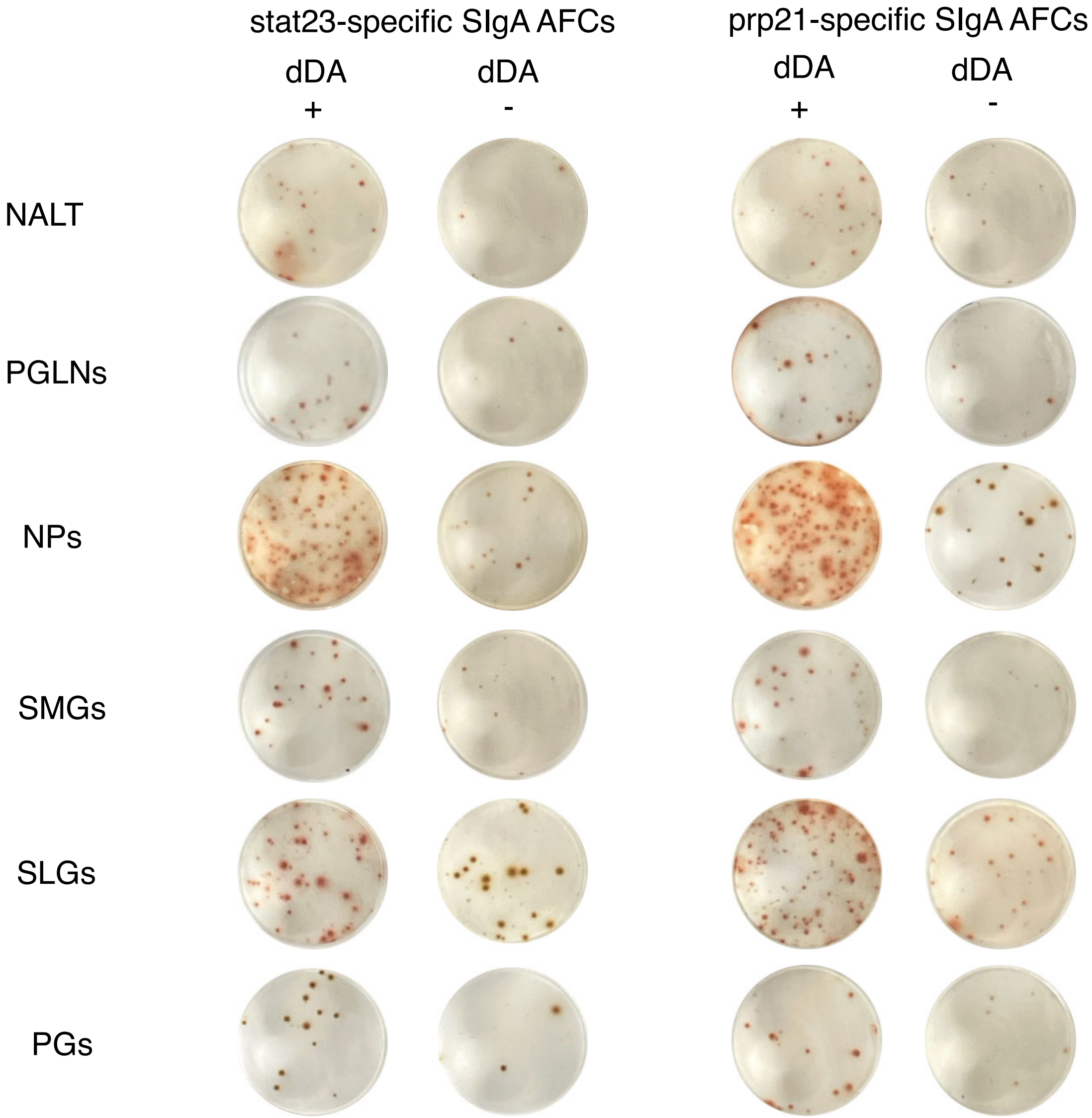

Fig. S1 The representative Ag-specific SIgA AFCs on the membranes of ELISPOT plates from NALT, PGLNs, NPs, SMGs, SLGs and PGs in mice given nasally double Ags with/without dDA. Mononuclear cells from NALT, PGLNs, NPs, SMGs, SLGs and PGs were subjected to ELISPOT assay to detect numbers of Ag-specific AFCs. Ninety-six-well nitrocellulose plates (Millipore) were coated with each Ag, incubated for 20 h at 4 ℃ and then washed extensively and blocked with 2% BSA in PBS solution. The blocking solution was discarded, and mononuclear cells (10^6 / well) were added to wells and incubated for 4 h at 37 ℃ in 5% CO_2 in moist air. Goat horseradish peroxidase-conjugated anti-mouse IgA Ab was used as detection Ab. Following overnight incubation, plates were developed by adding 3-amino-9-ethylcarbazole dissolved in 0.1M sodium acetate buffer containing H_2O_2 to each well, and AFCs were counted with the aid of a stereomicroscope.
