# Supplementary material for: Human salivary protein-derived peptides specific-salivary SIgA antibodies enhanced by nasal double DNA adjuvant in mice play an essential role in preventing Porphyromonas gingivalis colonization: an in-vitro study
Source: BMC Oral Health. 2023 Feb 24;23:123. doi: 10.1186/s12903-023-02821-6 (PMC9950703; doi:10.1186/s12903-023-02821-6)
Supplement: Supplementary file 2 — Additional file 2: Table S1. Proportions of CD11c+ DCs in mucosal lymphoid tissues of mice given nasal double Ags with/without dDA, as assessed by flow cytometry assay. Mice were nasally administered weekly for four consecutive weeks with the mixture of stat23 and prp21 with/without dDA. One week after the last immunization, the mononuclear cells from NALT, SMLNs, NPs, and three major salivary glands were collected and were stained with Brilliant violet 421-labeled anti-mouse CD11c monoclonal Ab. The resulting cells were subjected to flow cytometry analysis by FACSVerse®. The values are presented as the means ± SE of 10 mice in each group. Comparison were performed using a two-tailed unpaired Student’s t-test vs. mice immunized with the double Ags alone [dDA(-)], *p<0.05. [file 12903_2023_2821_MOESM2_ESM.pdf]

**Table S1 Proportions of CD11c$^+$ DCs in mucosal lymphoid tissues of mice given nasal double Ags with/without dDA, as assessed by flow cytometry assay.**

| | % of CD11c$^+$ cells in total lymphocytes | | | | | |
|---|---|---|---|---|---|---|
| dDA | NALT | PGLNs | NPs | SMGs | SLGs | PGs |
| + | *6.1 ± 1.8 | * 4.6 ± 1.1 | *9.5 ± 2.1 | 1.6 ± 0.7 | 1.8 ± 0.8 | 1.5 ± 0.8 |
| - | 1.3 ± 0.5 | 0.8 ± 0.4 | 3.6 ± 1.0 | 1.1 ± 0.6 | 1.5 ± 0.8 | 1.2 ± 0.6 |

Mice were nasally administered weekly for four consecutive weeks with the mixture of stat23 and prp21 with/without dDA. One week after the last immunization, the mononuclear cells from NALT, SMLNs, NPs, and three major salivary glands were collected and were stained with Brilliant violet 421-labeled anti-mouse CD11c monoclonal Ab. The resulting cells were subjected to flow cytometry analysis by FACSVerse®. The values are presented as the means ± SE of 10 mice in each group. Comparison were performed using a two-tailed unpaired Student's t-test vs. mice immunized with the double Ags alone [dDA(-)], * $p < 0.05$.
