# Supplementary material for: Human salivary protein-derived peptides specific-salivary SIgA antibodies enhanced by nasal double DNA adjuvant in mice play an essential role in preventing Porphyromonas gingivalis colonization: an in-vitro study
Source: BMC Oral Health. 2023 Feb 24;23:123. doi: 10.1186/s12903-023-02821-6 (PMC9950703; doi:10.1186/s12903-023-02821-6)
Supplement: Supplementary file 3 — Additional file 3 : Fig. S2. The typical FACS plot and gating strategy in NALT, PGLNSs and NPs. In FACS analysis, mononuclear cells from NALT, PGLNs and NPs were gated to lymphocytes by using the forward-and side-scatter properties and were subsequently analyzed for the populations of CD11c+ cells. Mononuclear cells from NALT, PGLNs, and NPs were stained with Brilliant violet 421-conjugated anti-mouse CD11c monoclonal antibody and were subjected to flow cytometric analysis by FACSVerse®. The graph represents typical profiles for each experimental group, and the percentages of CD11c+ DCs in the mononuclear cells are indicated in each graph. The dotted line indicates no stain samples. [file 12903_2023_2821_MOESM3_ESM.pdf]

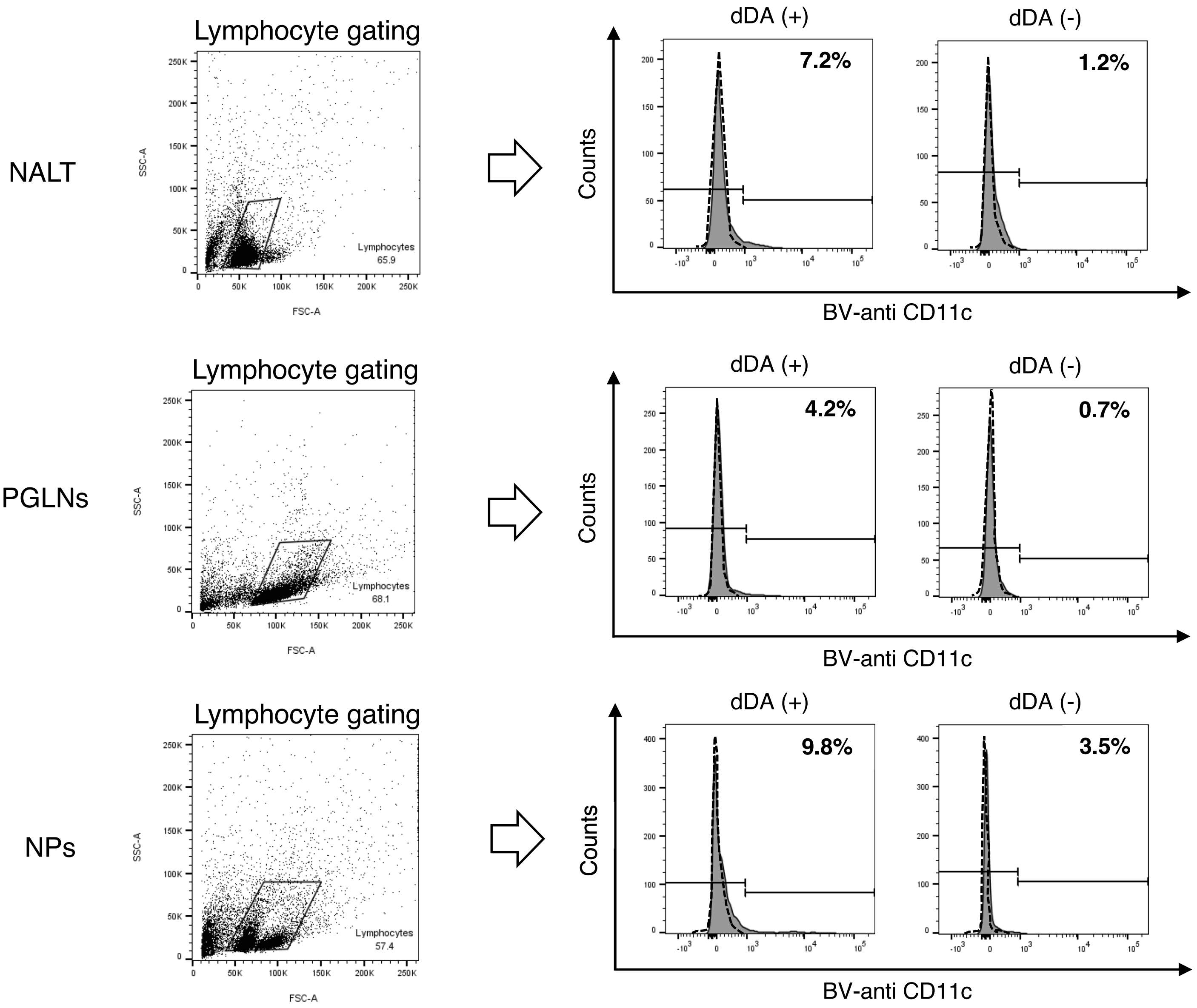

Fig. S2 The typical FACS plot and gating strategy in NALT, PGLNSs and NPs. In FACS analysis, mononuclear cells from NALT, PGLNs and NPs were gated to lymphocytes by using the forward-and side-scatter properties and were subsequently analyzed for the populations of CD11c$^+$ cells. Mononuclear cells from NALT, PGLNs, and NPs were stained with Brilliant violet 421-conjugated anti-mouse CD11c monoclonal antibody and were subjected to flow cytometric analysis by FACSVerse®. The graph represents typical profiles for each experimental group, and the percentages of CD11c$^+$ DCs in the mononuclear cells are indicated in each graph. The dotted line indicates no stain samples.
