# Supplementary material for: Human salivary protein-derived peptides specific-salivary SIgA antibodies enhanced by nasal double DNA adjuvant in mice play an essential role in preventing Porphyromonas gingivalis colonization: an in-vitro study
Source: BMC Oral Health. 2023 Feb 24;23:123. doi: 10.1186/s12903-023-02821-6 (PMC9950703; doi:10.1186/s12903-023-02821-6)
Supplement: Supplementary file 4 — Additional file 4: Table S2. stat23 and prp21-induced CD4+ Th1- and Th2-type cytokines from mice given nasal double Ags with/without dDA. Mice were nasally immunized weekly for 4 consecutive weeks with the mixture of stat23 and prp21 with/without dDA. One week after the final administration, CD4 T cells (4 x 10^6^ cells/mL) from NALT, PGLNs and NPs were cultured with stat23 and prp21 (each 1 μg/mL) in the presence of T cell-depleted splenic feeder cells (8 x 10^6^ cells/mL). The culture supernatants were harvested after 5 days incubation and analyzed by the respective cytokine-specific ELISA. The levels of each cytokine are expressed by subtracting the protein value of non-stimulated cultures from that of stimulated cultures. The values are presented as the means ± SE of three independent experiments. Each group consists of five mice. Comparisons were performed using a two-tailed unpaired Student’s t-test vs. mice immunized with double Ags [dDA(-)], *p<0.05. [file 12903_2023_2821_MOESM4_ESM.pdf]

Table S2 stat23 and prp21-induced $CD4^+$ Th1- and Th2-type cytokines from mice given nasal double Ags with/without dDA.

Mucosal inductive and effective tissues	dDA	IFN-γ (ng/mL)	IL-2 (pg/mL)	IL-4 (pg/mL)	IL-5 (pg/mL)
NALT	+	*2.4 ± 0.5	*86 ± 24	*38 ± 11	* 45 ± 14
	-	0.5 ± 0.4	34 ± 19	12 ± 6.3	18 ± 9.5
PGLNs	+	*1.5 ± 0.6	*42 ± 10	*25 ± 0.6	37 ± 12
	-	0.4 ± 0.4	16 ± 5.6	5.6 ± 2.5	29 ± 11
NPs	+	*1.6 ± 0.6	*62 ± 9.3	*40 ± 11	*27 ± 14
	-	0.8 ± 0.4	19 ± 12	15 ± 8.6	10 ± 6.8

Mice were nasally immunized weekly for 4 consecutive weeks with the mixture of stat23 and prp21 with/without dDA. One week after the final administration, CD4 T cells (4×10^6 cells/mL) from NALT, PGLNs and NPs were cultured with stat23 and prp21 (each 1 μg/mL) in the presence of T cell-depleted splenic feeder cells (8×10^6 cells/mL). The culture supernatants were harvested after 5 days incubation and analyzed by the respective cytokine-specific ELISA. The levels of each cytokine are expressed by subtracting the protein value of non-stimulated cultures from that of stimulated cultures. The values are presented as the means ± SE of three independent experiments. Each group consists of five mice. Comparisons were performed using a two-tailed unpaired Student's t-test vs. mice immunized with double Ags [dDA(-)], *$p < 0.05$.
